# A clinician's perspective of the role of renal sympathetic nerves in hypertension

**DOI:** 10.3389/fphys.2015.00075

**Published:** 2015-03-25

**Authors:** Alexandros Briasoulis, George L. Bakris

**Affiliations:** American Society of Hypertension Comprehensive Hypertension Center, Department of Medicine, The University of Chicago MedicineChicago, IL, USA

**Keywords:** resistant hypertension, renal denervation therapy

## Abstract

The renal sympathetic nerves have significant contribution to the control of different aspects of kidney function. Early animal studies of renal denervation in a large number of different models of hypertension showed that that RDN improved BP control. Recently, data from prospective cohorts and randomized studies showed that renal denervation therapy (RDN) is a safe procedure but is associated with only modest reduction of ambulatory blood pressure (BP) in patients on intensive medical therapy. The main goal of this article is to review the results of preclinical and clinical studies on the contribution of the renal sympathetic nervous system to hypertension and the therapeutic applications of catheter-based renal denervation.

## Introduction

Sympathetic activation participates in the development of the hypertension, by promoting the initial blood pressure elevation in the early clinical stages of the disease, and maintaining the blood pressure elevation (Johns et al., 2011). The adrenergic overdrive triggers not only elevations in blood pressure but contributes over time to end-organ damage and metabolic abnormalities detected in hypertensive patients.

Four decades ago Muller and Barajas (1972) reported the anatomical basis for a direct action of the sympathetic nervous system on renal tubular function by showing that norepinephrine-containing renal sympathetic nerve terminals are in direct contact with the basal membrane of all renal tubular segments, suggesting that renal sympathetic nerve activity can regulate renal tubular transport function. Indeed, the efferent and afferent renal nerves convey sensory stimuli between the sympathetic system and the kidney, thus providing a control system for the regulation of renal function (DiBona and Kopp, 1997). Under normal conditions, the renal sympathetic nerves regulate sodium homeostasis and participate in the arterial blood pressure (BP) control. In hypertensives, pathological alterations of this system contribute to abnormalities in sodium reabsorption and poorly controlled BP.

Despite the success of drug therapy in treating HTN and reducing associated adverse cardiovascular effects, the percentage of patients achieving adequate BP control worldwide remains low. According to the National Health and Nutrition Examination Survey (NHANES) dataset (Egan et al., 2011; Persell, 2011) the prevalence of resistant hypertension (RH) was 8.9% in hypertensives and 12.8% in treated hypertensives. In randomized controlled trials one third of patients did not achieve BP targets despite receiving ≥3 antihypertensive agents (Cushman et al., 2002; Gupta et al., 2011). In a recent report of data from the Kaiser Permanente health care systems, the incidence of RH was 1.9% within 1.5 years (Daugherty et al., 2012) and was related to increased cardiovascular and renal events.

The complex renal sympathetic system recently became of interest as renal sympathetic denervation treatment (RDN) was introduced into the treatment of patients with RH. Early uncontrolled cohort studies of patients with RH confirmed the safety of the procedure and reported substantial office BP reduction (Krum et al., 2009, 2013; Symplicity HTN-2 Investigators, 2010; Esler et al., 2014). However, evidence derived from the first randomized controlled trial Simplicity HTN-3 (Bhatt et al., 2014), failed to meet its primary efficacy end point, suggesting that RDN does not significantly lower office or ambulatory BP compared to medical therapy. The goal of this article is to review the role of the sympathetic renal nervous system in hypertension, in light of the recently reported results of RDN in patients with RH.

## Anatomy and physiology of the renal sympathetic nerves and implications in blood pressure control

The neural pathways for the sympathetic innervation of the kidney originate from the intermediolateral column of the spinal cord. Preganglionic fibers connect to ganglia along the sympathetic chain, and the splachnic ganglia including the paravertebral aortorenal ganglia (DiBona and Kopp, 1997). Within the ganglia, the preganglionic fibers connect with postganglionic neurons that then project to the kidney. Sympathetic outflow to the kidney is controlled by neural projections from brain nuclei such as the rostral ventrolateral medulla (RVLM), to intermediolateral column region of the spinal cord. Afferent sensory information arising from the renal sympathetic system travels to the nucleus tractus solitarius (NTS) where the central integration begins and as a result pathways are activated that track to the caudal ventrolateral medulla and RVLM.

The sympathetic nerves pass from the aortorenal ganglia and come in proximity with the renal artery to enter the kidney at the hilus after which they divide into smaller bundles in parallel with the divisions of the arterial circulation. As the nerves traverse deeper into the kidney, they begin to divide further and to form a network of fibers that penetrate throughout the cortex, juxtamedullary regions, and to a lesser extent in the medulla (Fazan et al., 2002). Each renal nerve bundle contains approximately 900 fibers. The vast majority of postganglionic sympathetic nerve fibers entering the kidney are unmyelinated with variable diameters (Sato et al., 2006). In human and animal models the maximal mean number of nerves was observed in the proximal and middle segments of the renal artery, whereas the least average number of nerves was seen in the distal segment. The circumferential distribution was greatest in the ventral and least in the dorsal regions (Tellez et al., 2013; Sakakura et al., 2014). In the main renal artery, distribution of the distance of nerves from the renal arterial lumen varied considerably, from <1 mm to >10 mm; however, the 75th percentile of the distance was 4.28 mm. Interestingly, 20% of hypertensive patients have additional small accessory renal arteries which also have sympathetic nerves (Sakakura et al., 2014).

The primary neurotransmitter released by the renal sympathetic nerves is norepinephrine. Stimulation of the renal sympathetic nerves increases norepinephrine production that results in increased sodium reabsorption by the renal tubular epithelial cells, contraction of smooth muscle cells (Esler et al., 2003), and renin release by the granular cells of the juxtaglomerular apparatus (Kopp et al., 1980).

Afferent nerve fibers are also found intrarenally in close vicinity to efferent sympathetic nerve fibers mainly projecting from the renal pelvis to the first neuron in the dorsal root ganglion (Stella and Zanchetti, 1991). Afferent nerves are less abundant compared to efferent and their proportion is not different between the proximal, middle, and distal segments. The peripheral axons of afferent renal sensory nerves may release substance P and calcitonin gene-related peptide as primary sensory neurotransmitters (Kopp et al., 2001). They exert an inhibitory effect on both ipsilateral and contralateral efferent renal sympathetic nerve activity. Inhibitory renorenal reflexes regulate of arterial pressure and sodium balance in normotensive healthy individuals leading to decreased afferent renal sympathetic nerve activity (Kopp et al., 2009). In various pathological conditions, activation of the afferent renal sensory nerves and the inhibitory renorenal reflexes are impaired. In these conditions, the excitatory renorenal reflexes will contribute to increased sodium retention and arterial pressure (Kopp et al., 2009).

Under normal quiet and unstressed conditions the level of renal sympathetic nerve activity does not affect renal blood flow. However, in states of anxiety and tension or in pathophysiological states, renal sympathetic nerve activity is sufficiently elevated so as to increase renal vascular resistance and decrease renal blood flow (Yoshimoto et al., 2004).

The activation of the renal sympathetic fibers has several effects. Firstly, efferent renal sympathetic activation decreases renal blood flow and glomerular filtration rate via contraction of the preglomerular smooth muscle cells. Secondly, it stimulates the release of norepinephrine from renal sympathetic nerves' terminals leading to direct activation of the postsynaptic alpha-1 adrenoceptors located on the renal tubular epithelial cells and the activation of beta-1 adrenoceptors on juxtaglomerular granular cells (Pettinger et al., 1985). The activation of beta1-adrenoreceptors on juxtaglomerular granular cells increases renin secretion rate, the stimulation of alpha1b-adrenoreceptors on renal tubular epithelial cells increases renal tubular sodium reabsorption, and the stimulation of alpha1a-adrenoreceptors on the renal arterial resistance vessels decreases renal blood flow (Pettinger et al., 1985; Plato, 2001).

The importance of increased renal sympathetic nerve activity in the development of hypertension was supported by the finding that renal denervation in a large number of different experimental animal models of hypertension either prevented, delayed the onset, or reduced the magnitude of the hypertension (Bonjour et al., 1969; Hesse and Johns, 1984; Kompanowska-Jezierska et al., 2001; Yoshimoto et al., 2004; Schlaich et al., 2009; Salman et al., 2010). Bonjour et al., showed that renal denervation in anesthetized dogs (Bonjour et al., 1969), increased in urinary flow rate and sodium excretion while neither renal blood flow nor glomerular filtration rate changed. They concluded that this increased output of water and sodium was due to the withdrawal of a direct action of the renal sympathetic nerves acting on renal tubules. Different groups showed that renal denervation decreases sodium and water reabsorption in all tubular segments including the proximal tubules, the loop of Henle and the distal convoluted tubule (Bello-Reuss et al., 1975, 1977). Additionally, renal denervation blunts the ability of the kidney to increase renin secretion in response to normal renin releasing stimuli (Johns, 1985).

Also, the activation of the renal afferent nerves contributes directly to systemic hypertension by modulating central sympathetic nervous system activity and promoting vasopressin and oxytocin release from the neuro-hypophysis (Echtenkamp and Dandridge, 1989). Patients early in the course of essential hypertension often have been demonstrated to have increased efferent sympathetic activity to the kidneys (Katholi, 1983). On the other hand, patients with essential hypertension with chronic kidney disease have been found to have increased centrally mediated sympathetic activity, possibly mediated by increased afferent renal sensory nerve activity (Hausberg et al., 2002).

Based on results from animal models of hypertension, denervation of efferent nerves can reduce renin release and sodium retention, improve renal blood flow, and facilitate blood pressure control (Holmer et al., 1994) while the denervation of afferent sensory nerves could attenuate the kidneys' contribution to centrally mediated sympathetic nervous system activity (Katholi et al., 1983, 1984).

## Renal denervation therapy for patients with treatment resistant hypertension

Surgical renal denervation has been studied in humans for the treatment of resistant hypertension and shown effective for reducing sympathetic outflow to the kidneys, and renin release, without adversely affecting other functions of the kidney such as glomerular filtration rate (GFR) and RBF. However, these surgical approaches were frequently complicated by severe orthostatic hypotension, and urinary incontinence impotence (Smithwick and Thompson, 1953).

Renal sympathetic denervation treatment (RDT) using a radiofrequency ablation catheter presents several significant advantages over surgical approaches targeting the renal sympathetic nerves. It is a localized procedure, it is minimally invasive, it has no systematic side effects, and its procedural and recovery times are very short. The Symplicity Renal Denervation System and newer multielectrode catheters comprise of endovascular energy delivery catheters and an automated radiofrequency generator. Once in place within the renal artery, the tip of the catheter is placed against the arterial wall in several places where it delivers radiofrequency energy to the surrounding sympathetic nerves according to a proprietary, computer-controlled algorithm. Typical procedure starts distally in the renal artery with the catheter being withdrawn by pulling and rotating the tip, and it involves at least 4 focal treatments with a distance of ≥5 mm between each site (Krum et al., 2009; Symplicity HTN-2 Investigators, 2010). Renal sympathetic nerves are more abundant in the superior area of the arterial ostium. Recent studies have shown that in the proximal segments of renal artery these nerved are localized >5 mm from the lumen, a distance which may be beyond the ablation depth of currently used catheters which is approximately 3–4 mm (Tzafriri et al., 2014).

### Early clinical studies of RDN

The Symplicity HTN-1 study (Krum et al., 2009) assessed safety of RDN. Short-term repeat angiography and 6-month magnetic resonance angiography, available for 34 patients, revealed no residual luminal irregularities at any treatment site. The effectiveness of RDN was confirmed by renal norepinephrine (NE) spillover. This assay confirmed a significant mean post-treatment reduction in renal norepinephrine spillover of 47% in 10 randomly selected patients. In this cohort study, RDN lowered office systolic blood pressure by 27 mm Hg at 12 months, and 85% of the patients responded to therapy with a reduction of systolic blood pressure exceeding 10 mm Hg (Krum et al., 2009). Importantly, six of the 45 patients who underwent catheter-based renal denervation had office systolic blood pressure reductions of less than 10 mm Hg and were non-responders.

At 36 months office BP was reduced by an average of 32/14 mmHg in 88 patients with complete data with 6 non-responders only (Krum et al., 2013). Although, striking and sustained BP reductions were seen, ambulatory blood pressure monitoring was not used during follow-up and allowed medication adjustments during this period. After three years of follow-up of Symplicity HTN-1 there was still no indication that the number of antihypertensive medications could be reduced by RDN (Krum et al., 2013).

A recent larger prospective uncontrolled study specifically examined the BP response to RDN as measured by ambulatory BP monitoring (Mahfoud et al., 2013). In 346 subjects who underwent RDN following the Symplicity HTN-2 protocol were followed for up to 12 months, there was a significant reduction in 24-h systolic BP (−12 mm Hg) and diastolic BP (−7 mm Hg) at 12 months which was much smaller than the reported office SBP and DBP reduction. Both these studies had important limitations. Apart from being nonrandomized and uncontrolled, a high rate of subjects were lost to follow-up. In the study by Mahfoud et al (Kopp et al., 1980) the significant discrepancy between office BP and ambulatory BP reduction may have been due to large bias in office BP measurements.

The Symplicity HTN-2 multicenter, prospective, randomized trial (Symplicity HTN-2 Investigators, 2010) assessed the safety and change in office BP in 106 patients with RH. The inclusion criteria were the same with the first proof-of-concept study. Office BP was reduced by 32/12 mm Hg in the renal denervation group, but did not differ from baseline in the control group. Similar differences in home BP were seen between the two groups were observed. Also, RDN reduced BP during exercise without compromising chronotropic competence in patients with resistant hypertension (Ukena et al., 2011). There were no serious complications related to the device or procedure. The results of the 3-year follow-up analysis reported a pronounced sustained office SBP and DBP reduction with approximately 15% nonresponders and not substantial reduction in mean number of medications (Esler et al., 2014). Despite the limited follow-up time, number of patients and lack of ambulatory BP this study showed a significant reduction in office BP can be safely achieved with catheter-based RDN in patients with resistant hypertension.

### Symplicity HTN-3 and recent studies (Table 1)

The Symplicity HTN-3 (Bhatt et al., 2014) randomized 535 patients with resistant essential hypertension and an estimated glomerular filtration rate above 45 mL/min/1.73 m^2^ to undergo renal denervation with previous treatment or to maintain previous treatment alone. At 6 months, the decrease in office and ambulatory systolic BP in the RDN group was a mean of 14.13 and 7 mm Hg respectively compared with a fall of 11.74 and 5 mm Hg in the control group. Neither of these differences in BP met the prespecified criteria for statistically significant superiority. Interestingly, the 7 mm Hg decrease in ambulatory systolic BP after RDN was similar to reduction in 24-h ambulatory systolic BP seen in the 12 patients in the Symplicity HTN-1 study (Krum et al., 2013) but less pronounced compared to the 11 mmHg difference seen in 20 patients from Symplicity HTN-2 (Symplicity HTN-2 Investigators, 2010). In the pre-specified subgroup analysis, office SBP was significantly reduced by RDN in the non-African American patients and those younger than age 60, but this was not translated into meaningful difference in ambulatory BP measurements (Bakris et al., 2014).

**Table 1:** **Prospective randomized controlled trials of renal denervation therapy in resistant Hypertension**.

**Studies**	**Sample size (RDN/Controls)**	**Age**	**Number of antihypertensives**	**Catheter type**	**Method of BP measurement**	**ABPM difference from baseline (mmHg)**	**Office SBP/DBP difference from baseline (mmHg)**	**Follow-up (months)**	**Country**
Symplicity-HTN 3, Bhatt et al., 2014	364/171	57.9 ± 10.4	5.1 ± 1.4	Symplicity	Office BP ABPM	RDN: −7/−4 Control:−6/−3	RDN: −14/−7 Control: −12/−5	6	USA
Rosa et al., 2014	52/54	56 ± 12	5.1 ± 1.2	Symplicity	Office BP ABPM	RDN: −9/−5 Control:−9/−4	RDN: −12/−7 Control: −14/−7	6	Czech Republic
Fadl Elmula et al., 2014	9/10	57 ± 10.9	5.1 ± 1.6	Symplicity	Office BP ABPM	RDN: −10/−7 Control:−19/−11	RDN: −12/−2 Control: −28/−11	6	Norway
Symplicity HTN-2 Investigators, 2010; Esler et al., 2014	52/54	58 ± 12	5.2 ± 1.5	Symplicity	Office BP ABPM	RDN: −11/−7 Control: −3/−1	RDN: −32/−12 Control: 1/0	6	Australia, Europe
Ukena et al., 2011	37/9	59 ± 9.4	5.9 ± 1.4	Symplicity	Office BP	-	RDN:−31/−9 Control:0/1	3	Germany
Pokushalov et al., 2012	13/14	56.5 ± 9	3.8 ± 0.4	Navistar/ThermoCool	Office BP	-	RDN:−15/−7 Control: −10/−2	12	Russia, USA

BP, Blood Pressure; ABPM, Ambulatory Blood Pressure Monitoring; HTN, Hypertension; RDN, Renal Denervation Therapy.

The major discrepancies between Symplicity HTN-3 and previous studies may be in part attributed to baseline population differences and selection bias. Symplicity HTN-3 included more obese patients, of African-American decent, at higher cardiovascular risk, treated with diuretics and aldosterone antagonists more frequently compared to Symplicity HTN-2. The Symplicity HTN-3 investigators may have been less experienced than the Symplicity HTN-1 and 2 investigators. Additionally, the selection of patients only based on elevated office BP in Symplicity HTN-1 and HTN-2 may have resulted in selection bias due to lack of standardization and substantial variability of BP affected by the regression to the mean phenomenon.

A more recent European cohort study of 109 patients with RH and a prospective uncontrolled trial of a new multielectrode catheter confirmed the disparities between office and ambulatory measurements and showed modest reduction of ambulatory BP (Worthley et al., 2013; Persu et al., 2014). In contrast to the disappointing reports of Symplicity HTN-3 and subsequent small studies, the recently reported Global SYMPLICITY Registry (Böhm et al., 2014) of 1000 consecutively enrolled patients not only confirmed the safety of RDN but also suggested that RDN lowers office and ambulatory BPs at 6 months. It is also noteworthy to mention that beyond BP reduction, RDN has also been shown to be effective in the treatment of other conditions coexisting with resistant hypertension such as impaired glucose tolerance (Mahfoud et al., 2011; Witkowski et al., 2011), obstructive sleep apnea severity (Witkowski et al., 2011), and left ventricular hypertrophy (Brandt et al., 2012). Brandt et al examined the effect of RDN on diastolic function and LVH in patients with resistant hypertension. Besides reduction of systolic and diastolic blood pressure at 1 and 6 months, similar to the effect observed in the Symplicity-2 HTN trial, RDN significantly reduced LVH and improved E/E' prime ratio and isovolumetric relaxation time as well as systolic LV function (Brandt et al., 2012). Notably, in 5 non-responders LV mass index was significantly decreased while in 4 non-responders the diastolic function was significantly improved, indicating BP-independent effects of RD on LVH and diastolic dysfunction. More recently, Mahfoud et al demonstrated a decrease in LV mass index, as assessed by using cardiac Magnetic Resonce, in both responders and non-responders undergoing RDN (Mahfoud et al., 2014). The beneficial effects of RDN on LV mass independently of BP reduction were confirmed by Doltra et al in 23 patients undergoing RDN who exhibited reduction of LV mass not exclusively due to a reversion of myocyte hypertrophy but also to reduction of interstitial myocardial fibrosis (Doltra et al., 2014). It's presumed that these beneficial effects are linked to actions of RDN on renin-angiotensin-aldosterone axis and sympathic nervous system activity as discussed above.

However, the favorable metabolic effects of RDN were not confirmed by Symplicity HTN-3, which did not show any significant between-group difference in the change in glycated hemoglobin levels in the RDN group or in the subgroup of patients with diabetes (Bhatt et al., 2014).

**Figure 1:**
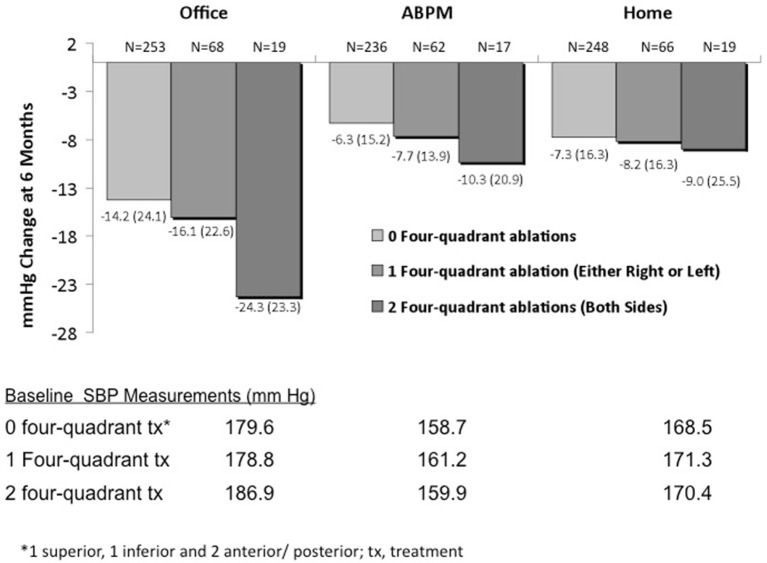
**Systolic blood pressure change at symplicity HTN-3 study at 6 months according to ablation pattern**.

Based on the findings of the first cohorts and randomized trials a number of concerns arise regarding the utility of RDN on patients with RH: (i) A limited number of patients with RH are candidates for the procedure due to presence of secondary form of HTN, CKD, normal home BP measurement or unsuitable anatomy. (ii) A significant portion of patients (15–30%) will have less than 10/5 mmHg BP reduction with RDT due to procedural-related limitations, operator experience and number of treatment delivered. In a subgroup analysis of Symplicity HTN-3 higher number of ablations (10–13) and also ablations in all for quadrants of the arterial wall cross sections (Figure 1) were associated with significant ambulatory BP reduction compared to the sham control group (Kandzari et al., 2015). (iii) Non-adherence to antihypertensive regimens affects more than 50% of patients with difficult to control hypertension (Jung et al., 2013). In Symplicity-HTN 3, appropriate combination and dosage of antihypertensive regimens, improved patient compliance and assessment with home and ambulatory BP led to substantial BP reduction in the control group which was greater compared to previous RDN trials. The importance of medication adherence and structured adjustment of antihypertensive medications was also shown in the recently published Oslo RDN trial (Fadl Elmula et al., 2014), which stopped early in view of the dramatic superiority of adjusted drug treatment and witnessed medication intake compared to RDN at 6 months of follow-up. (iv) Finally, RDN may not be suitable for all subgroups of patients regardless of the degree of sympathetic activity. In SYMPLICITY HTN-3 subgroup analysis revealed that African American control patients demonstrated an unusually greater decrease in systolic blood pressure compared with non-African American controls and a blunted response to RDN compared to non-African Americans. The marked reduction in blood pressure in the sham control group could be related to a change in medical adherence, type of therapy or degree of sympathetic activation (Kandzari et al., 2015).

## Conclusion

The renal sympathetic nerves have significant contribution to the control of different aspects of kidney function. Early animal studies of renal denervation in a large number of different models of hypertension showed that that RDN either prevented, delayed the onset, or reduced the magnitude of the hypertension. Additionally, the preclinical and clinical studies reviewed above, have provided comprehensive insight into the mechanisms that account for the BP lowering during suppression of renal sympathetic outflow and propose an alternative approach to improve BP control in patients with resistant hypertension. Future randomized trials should be performed in experienced centers using newer catheters and better designed techniques in carefully selected compliant patients on appropriate antihypertensive drug combinations in whom all other measures have failed.

### Conflict of interest statement

Professor Bakris is a consultant for Medtronic and Kona and served as co-principal investigator of SYMPLICITY HTN-3. The author declares that the research was conducted in the absence of any commercial or financial relationships that could be construed as a potential conflict of interest.
